# Resting-state functional connectivity disruption between the left and right pallidum as a biomarker for subthreshold depression

**DOI:** 10.1038/s41598-023-33077-3

**Published:** 2023-04-18

**Authors:** Yosuke Sato, Go Okada, Satoshi Yokoyama, Naho Ichikawa, Masahiro Takamura, Yuki Mitsuyama, Ayaka Shimizu, Eri Itai, Hotaka Shinzato, Mitsuo Kawato, Noriaki Yahata, Yasumasa Okamoto

**Affiliations:** 1 Department of Psychiatry and Neurosciences, Hiroshima University, 1-2-3 Kasumi, Minami-ku, Hiroshima, 734-8551 Japan; 2 Deloitte Analytics R&D, Deloitte Touche Tohmatsu LLC, Tokyo, Japan; 3 Department of Neurology, Shimane University, Matsue, Japan; 4 Center for Brain, Mind and KANSEI Research Sciences, Hiroshima University, Hiroshima, Japan; 5 ATR Brain Information Communication Research Laboratory Group, Kyoto, Japan; 6 Institute for Quantum Life Science, National Institutes for Quantum Science and Technology, Chiba, Japan

**Keywords:** Diagnostic markers, Depression

## Abstract

Although the identification of late adolescents with subthreshold depression (StD) may provide a basis for developing effective interventions that could lead to a reduction in the prevalence of StD and prevent the development of major depressive disorder, knowledge about the neural basis of StD remains limited. The purpose of this study was to develop a generalizable classifier for StD and to shed light on the underlying neural mechanisms of StD in late adolescents. Resting-state functional magnetic resonance imaging data of 91 individuals (30 StD subjects, 61 healthy controls) were included to build an StD classifier, and eight functional connections were selected by using the combination of two machine learning algorithms. We applied this biomarker to an independent cohort (*n* = 43) and confirmed that it showed generalization performance (area under the curve = 0.84/0.75 for the training/test datasets). Moreover, the most important functional connection was between the left and right pallidum, which may be related to clinically important dysfunctions in subjects with StD such as anhedonia and hyposensitivity to rewards. Investigation of whether modulation of the identified functional connections can be an effective treatment for StD may be an important topic of future research.

## Introduction

Recently, there has been growing interest in subthreshold depression (StD)^1^, which is defined as clinically relevant depressive symptoms not meeting the criteria for full-blown major depressive disorder (MDD)^2^. StD is highly prevalent among university students worldwide^3^ and its prevalence seems to be increasing^4^. Late adolescent university students are reportedly vulnerable to depression and anxiety^5–7^, have a higher rate of depression compared to the general population^8^, and their depressive symptoms may be related to an increased risk for suicidal ideation and suicide attempts^9^. StD is associated with severe functional impairments and has a negative impact on the quality of life. In addition, StD is a risk factor for developing MDD^6,10^. Hence, the identification of late adolescents with StD may provide a basis for developing effective interventions that could lead to a reduction in the prevalence of StD and prevent the development of MDD. However, knowledge about the neural basis of StD remains limited, which makes it challenging to develop effective biomarkers and treatments^11^.

Some studies have evaluated impaired brain regions and abnormal patterns of brain connectivity to develop novel biomarkers for StD using resting-state functional magnetic resonance imaging (rs-fMRI)^11–18^. In early studies, it was reported that spontaneous neuronal activity, as measured by the amplitude of low-frequency fluctuations, is altered, that resting-state functional connections (FCs) are impaired, and that subcortical degree centrality is decreased and cortical degree centrality is increased in subjects with StD^12,13,16^. In studies pertaining to brain networks, it was suggested that FCs between subregions of the anterior cingulate cortex are altered, that the FCs of the habenula within the default model network are increased, and that the FCs within salience networks are diminished in individuals with StD^11,17^. Recently, beyond the group-level analyses used in these previous studies, a data-driven approach using machine learning, which identifies phenotypes in a way that is clinically useful and can be applied to clinical diagnosis or prognosis, has gained attention^19^. In a recent investigation, by using the Support Vector Machine, which has been used frequently to identify imaging biomarkers in some diseases^20^, connectome-based biomarkers predicting StD associated with regions of the thalamus were identified, but with an insufficient generalization capability^21^. If the generalization capability to independent data is not sufficient, it is difficult to discuss the pathophysiology of the target disease through FC features selected by machine learning. Yahata et al.^22^ developed an rs-fMRI-based classifier for autism spectrum disorder with generalization capability by combining two machine learning algorithms, L1-regularized sparse canonical correlation analysis (L1-SCCA)^23^ and sparse logistic regression (SLR)^24^, to overcome the two major difficulties causing poor generalization performance, i.e., over-fitting and nuisance variables, and additionally by testing the classifier in an independent validation cohort^22^. By using the same method, we also developed a classifier for MDD with melancholic features that were generalized to an independent cohort and extracted critically important FCs^25^. To our knowledge, no study has attempted to identify an rs-fMRI-based biomarker for StD and confirmed its generalization capability. Therefore, the purpose of the present study was to develop a generalizable classifier for StD in late adolescents by using the combination of two machine learning algorithms, L1-SCCA and SLR^22,25^. Another purpose of this study was to shed light on the underlying neural mechanisms of StD by using the critically important extracted FCs. In late adolescents, the StD group has been characterized only by hyposensitivity to environmental rewards in previous studies, whereas the MDD group has been characterized by a higher frequency of avoidance and hyposensitivity to environmental rewards compared with the non-depression group^26^. Moreover, anhedonia is one of the most common symptoms of StD^27^ and, in adolescents, predicts adult MDD^28^. In previous studies, reward dysfunction was observed in depression patients. Pizzagalli et al. found that MDD patients showed significantly weaker responses to gains in the striatum^29^. The abnormal resting-state FCs of the ventral striatum, a key region in the reward network, was observed in StD subjects^30^. Thus, we examined if the identified FCs in the StD classifier were associated with anhedonia and reward responsiveness in the present study.

## Results

### Highly accurate classifier for StD and generalization to an independent cohort

A classifier was constructed based on the FCs of each subject to distinguish StD subjects from HCs. The classification was first carried out by feature-selection from all 9316 FCs with L1-SCCA and SLR. Then, for this classifier, the weighted linear summation (or linear discriminant function) of the identified FCs was computed. This algorithm was applied to the training dataset (n = 91, Table 1), and then a classification accuracy of 80% (area under the curve 0.84, sensitivity 70%, specificity 85%; *p* = 0.001 with a permutation test, Supplementary Fig. S1a,c) was revealed by the leave-one-out cross validation (LOOCV) procedure. Next, the classifier was tested on an independent validation cohort (*n* = 43), and then a classification accuracy of 79% (area under the curve 0.75, sensitivity 84%, specificity 72%; *p* = 0.001 with a permutation test, Supplementary Fig. S1b,d) was revealed.

**Table 1:** Demographic and clinical information of the participants used to construct the subthreshold depression (StD) classifier.

	Training dataset			Test dataset		
	StD	Healthy controls	*p*-value	StD	Healthy controls	*p*-value
No. of participants	30	61	NA	16	27	NA
Sex, male/female	19/11	32/29	0.326	10/6	18/9	0.782
Age (years), mean (SD)	18.2 (0.4)	18.4 (0.5)	0.054	18.6 (0.7)	18.5 (0.6)	0.921
BDI-II, mean (SD)	16.8 (3.5)	3.0 (1.9)	0.000***	20 (5.0)	3 (2.2)	0.000***

The differences between StD subjects and healthy controls were tested by a two-tailed *t* test (for age and BDI-II) or the chi-squared test (for sex). ***p < 0.001. *BDI*-*II* Beck's Depression Inventory-II, *NA* not applicable, *SD* standard deviation.

### FCs in the classifier

**Figure 1:**
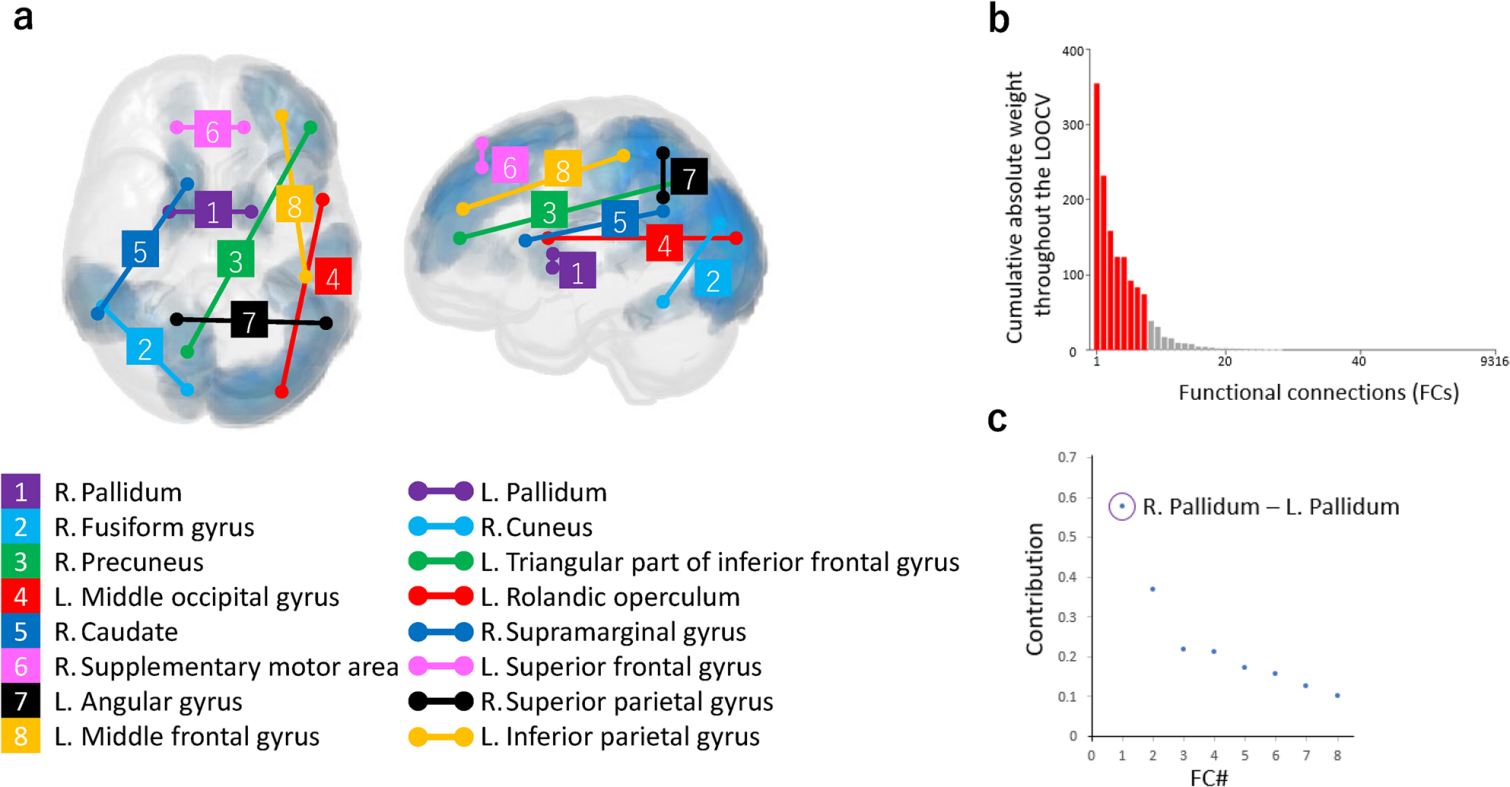
The eight identified functional connections (FCs) for the subthreshold depression (StD) classifier. (**a**) The 16 brain regions connected by the eight FCs of the StD biomarker (rendered with: MRIcroGL 64-bit, June 12, 2015, https://www.nitrc.org/projects/mricrogl). (**b**) Contribution of individual FCs to the StD biomarker. The cumulative absolute weights are shown for all 9316 FCs, of which 29 FCs were selected at least once throughout the leave-one-out cross validation process. The eight FCs identified through the StD classifier were derived from a key subset of the 29 FCs (the red columns represent the eight identified FCs, and the gray columns represent the remaining 21 FCs). (**c**) Contribution of each of the eight FCs to the StD biomarker.

Then, the neural basis of the StD classifier was investigated. The sparse classification algorithm allowed the survival of eight FCs from 29 FCs that were selected at least once throughout the LOOCV procedure based on their contribution to the classifier (Table 2 and Fig. 1a). The robustness and stability of the detected FCs throughout the cross-validation procedure are shown in Fig. 1b. Furthermore, we confirmed that the weights of the eight identified FCs across the LOOCV procedure were significantly nonzero (two-sided Wilcoxon signed rank test, *p* < 0.001), indicating their important contribution to the classifier. The contribution index of individual FCs to the corresponding connection, which was defined by the difference of each FC between StD subjects and HCs multiplied by the classifier weight, is plotted in Fig. 1c. We found that FC#1 (right pallidum to left pallidum) between inter-hemispheric regions showed the most outstanding contribution of the eight FCs. We also found that only FC#1, out of 9316 FCs, was identified as having a significant difference between StD and HCs by using conventional between-group comparison (two-sample *t* test with Bonfferoni correction, *p* < 0.05/9316).

**Table 2:** Characteristics of the eight interregional functional connections (FCs) used in the subthreshold depression (StD) classifier.

ID	Name	Lat	BrainVISA Sulci Atlas (sulcus)	BA	rStD	rHC	Weight	Contribution
1	Pallidum	R	Pallidum	–	0.344	0.513	− 3.41	0.578
	Pallidum	L	Pallidum	–				
2	Fusiform gyrus	R	Internal occipito-temporal lateral sulcus	37	0.147	0.341	− 1.90	0.370
	Cuneus	R	Cuneal sulcus	18				
3	Precuneus	R	Transverse parietal sulcus	7	− 0.200	− 0.287	2.51	0.217
	Triangular part of the inferior frontal gyrus	L	Anterior inferior frontal sulcus	45				
4	Middle occipital gyrus	L	Lobe occipital	19	− 0.149	− 0.038	− 1.91	0.213
	Rolandic operculum	L	Anterior sub-central ramus of the lateral fissure	48				
5	Caudate	R	Caudate	–	− 0.058	0.044	− 1.67	0.172
	Supramarginal gyrus	R	Anterior terminal ascending branch of the superior temporal sulcus	40				
6	Supplementary motor area	R	Median frontal sulcus	6	0.359	0.467	− 1.46	0.157
	Superior frontal gyrus	L	Median frontal sulcus	9				
7	Angular gyrus	L	Anterior terminal ascending branch of the superior temporal sulcus	39	− 0.153	− 0.297	0.88	0.127
	Superior parietal gyrus	R	Superior parietal sulcus	7				
8	Middle frontal gyrus	L	Intermediate frontal sulcus	46	− 0.061	0.016	− 1.33	0.102
	Inferior parietal gyrus	L	Superior postcentral intraparietal superior sulcus	40				

The mean functional correlation values for StD (rStD) and healthy controls (rHC) are presented for individual FCs. Since the StD group was the positive class in the classification of the StD and HC populations, the sign of weight was positive when rStD > rHC, whereas it was negative when rStD < rHC. Contribution was computed as (rStD − rHC) × weight. *BA* Brodmann area.

### Association of important FCs with anhedonia and reward responsiveness

**Figure 2:**
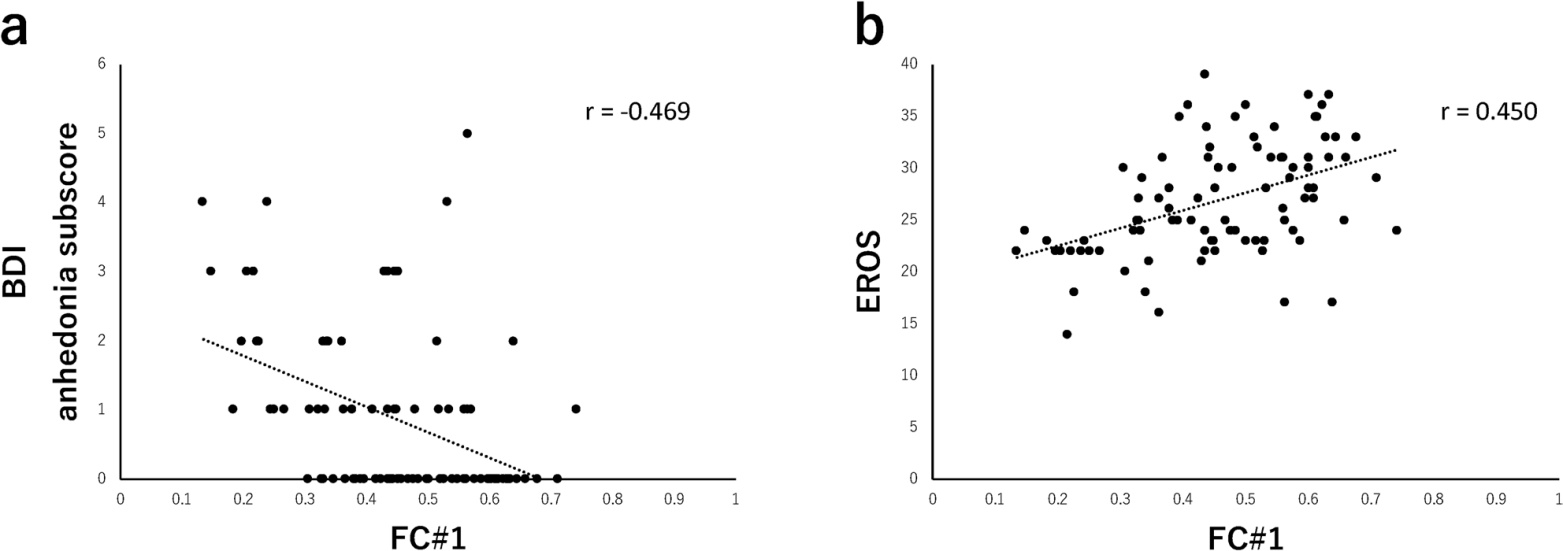
A scatter plot of correlations between the identified functional connections (FCs) and clinical scores. (**a**) FC#1 shows a negative correlation with the BDI anhedonic subscore (r = − 0.469, *p* < 0.006*). (**b**) FC#1 shows a positive correlation with EROS score (r = 0.450, *p* < 0.006*). *Bonferroni-adjusted significance level of *p* < 0.05/8 (= 0.006). *BDI* Beck's Depression Inventory, *EROS* Environmental Reward Observation Scale.

We evaluated the relevance of the important FCs to anhedonia and reward responsiveness, which are features of StD^31^. FC#1 and FC#2 had significant negative correlations with the BDI anhedonic subscore. FC#1 and FC#6 had significant positive correlations with the EROS score. Of the eight FCs, only FC#1 had significant correlations with the BDI anhedonic subscore and EROS score (Table 3, Fig. 2a,b). Also, similar results were obtained in the sum of training and test dataset (Supplementary Table S3).

**Table 3:** Relationship between each functional correlation value and clinical score.

Identified FCs	BDI anhedonic subscore		EROS	
	*r*	*p*	*r*	*p*
FC#1	− 0.469	0.000*	0.450	0.000*
FC#2	− 0.321	0.002*	0.282	0.007
FC#3	0.287	0.006	− 0.119	0.261
FC#4	− 0.264	0.011	0.183	0.083
FC#5	− 0.241	0.021	0.185	0.079
FC#6	− 0.256	0.014	0.383	0.000*
FC#7	0.199	0.059	− 0.217	0.039
FC#8	− 0.156	0.141	0.147	0.164

Clinical scores associated with anhedonia (BDI anhedonic subscore and EROS). *Bonferroni-adjusted significance level of *p* < 0.05/8 (= 0.006). *BDI* Beck's Depression Inventory, *EROS* Environmental Reward Observation Scale, *FC* functional connection.

## Discussion

Herein, we generated a neuroimaging-based biomarker for StD of which the generalization capability was confirmed in an independent validation cohort. Furthermore, our findings suggest that the FC between the left and right pallidum, which was the most important of the eight FCs identified for this classifier, may be associated with clinically important dysfunctions in subjects with StD such as hyposensitivity to rewards and anhedonia.

In recent years, some impaired brain regions and abnormal patterns of brain connectivity have been evaluated in subjects with StD by using rs-fMRI^11–18^. Unfortunately, these findings could not be applied to direct diagnosis, but they are useful for the identification of disease biomarkers associated with the dysfunctions observed in StD. This is because the individual predictive ability of these biomarkers was not evaluated, even though they were statistically significant at the group level. Following this, there has been increasing interest in using machine learning algorithms in order to predict phenotypes in a way that allows characterization at the level of the individual beyond group level analyses^19^. However, also in these studies, there were some limitations regarding the generalizability and feasibility of the identified markers, for example, there were too many predictive FCs to explain StD individually^21^. Yahata et al.^22^ overcame these limitations by combining two machine learning algorithms, L1-SCCA and SLR, and identified rs-fMRI-based classifiers for autism spectrum disorder with high generalization^22^. This technique was also used successfully to extract a few FCs related only to the core characteristics of melancholic MDD^25^. By using the same approach, we developed a generalizable rs-fMRI-based biomarker for StD tested on an independent validation cohort. The test data were acquired using a Siemens Verio scanner that was upgraded to a Skyra scanner including a retrofit of hardware and software. Imaging was also performed with a new protocol that had higher spatial and temporal resolution than used for the discovery data (Supplementary Table S1). Scanner specifications^32^ and imaging parameters^33^ are known to affect MRI data. Therefore, it is considered to be a great advantage of our biomarker that it showed generalization performance for different machines and different protocols without special harmonization.

Our findings demonstrated that FC#1 (right pallidum to left pallidum), which was the most important of the eight FCs identified for this classifier, may be associated with the clinically important dysfunctions in subjects with StD such as hyposensitivity to rewards and anhedonia. Of the eight identified FCs, FC#1 showed the strongest negative correlation with the BDI anhedonic subscore and strongest positive correlation with the EROS score. In addition, both of these correlations were still significant after multiple comparison corrections only for FC#1. These results suggest that the lower FC between the right and left pallidum may be relevant to hyposensitivity to rewards and anhedonia in subjects with StD. It is difficult to interpret that the FC between bilateral pallidum was not selected as an important feature to discriminate between MDD and healthy subjects in our previous study^25^. To verify whether the key findings of this study targeting StD also apply in MDD, we analyzed previous data (92 patients with MDD vs. 92 HCs from Ichikawa et al.^25^) focusing on the FC between bilateral pallidum. As a result, there was a trend toward decreased FC between bilateral pallidum in MDD compared to HCs, which was not significant (*p* = 0.067). It is unclear why the decreased FC between bilateral pallidum is more characteristic of StD than MDD, but it is possible that the balance of FC changes after onset. This issue needs further clarification in future longitudinal cohort studies.

The right and left pallidum are connected via the anterior commissure through the lateral nucleus of the globus pallidus^34,35^. The ventral pallidum reportedly plays a crucial role in motivation^36^. In rats and monkeys, electrical stimulation within the ventral pallidum supports self-stimulation behavior as a reward in and of itself^37,38^. In addition, in humans, activation in the ventral pallidum is associated with receiving food and money as a reward^39,40^. It has been suggested that the ventral pallidum is deeply involved in the regulation of motor action based on reward expectation^41^. Classically, lesions of the globus pallidus are reportedly related to anhedonia, decreased social interactions, and flattened affect^42,43^. Given that late adolescents with StD have been characterized by reward system dysfunction^26^, our findings suggest that the inter-hemispheric connectivity of the pallidum may be especially important in this process. Indeed, the volume of the bilateral pallidum is reportedly decreased and has a negative association with the severity of depressive symptoms in young StD subjects^44^.

Although the FC between the left and right pallidum was identified as a particularly important biomarker of StD, the other seven FCs contained areas in which abnormalities have been suggested in depression. In particular, the FC between the precuneus and the left triangular part of the inferior frontal gyrus is interesting because it is in the same direction as the connection between the precuneus and left dorsolateral prefrontal cortex, which was identified by Ichikawa et al. as an important connection in the biomarker for melancholic MDD^25^. Further research is needed in longitudinal studies, such as which connections are involved in the development of MDD.

There are four limitations related to the present study. First, because our study was cross-sectional, we cannot determine if these observations are the cause or result of StD. Additionally, the relatively mild depressive symptoms of StD subjects can change easily over time. Future longitudinal studies are required to address this question and will help further our understanding of the neural basis of StD. Second, the sample size of the current study was relatively small. The performance of a classifier is directly influenced by sample size, with which prediction accuracy may decrease^45^. Thus, a larger sample size is needed to validate the current findings further. Third, there were some differences in head motion between the two groups in the training dataset (Supplementary Table S2) that may influence the classifier performance, although six head motion parameters were linearly regressed out. Motion artifacts contribute substantially to the rs-fMRI signal and are not fully countered by motion regressions^58^. Some efforts may have to be directed at preventing head motion, such as behavioral or physical interventions^46^. Moreover, attention should be paid to the neurobiological basis of head motion as identified by Zeng et al.^47^. Differentiating true disease effects from the correlates of motion tendency is critical for using connectivity markers in the clinical area because correlates of motion may reduce specificity of biomarkers. Fourth, the cerebellum was not incorporated in the construction of the classifier because it was truncated in many participants' images. Cerebellar abnormalities have been the focus of increasing concern recently in depression^48^. There are several studies on StD subjects from rs-fMRI that has identified aberrant cerebellar activity^12,13^.

In summary, this study generated a generalizable rs-fMRI-based classifier for the accurate prediction of StD for the first time. For the StD classifier, the FC between the left and right pallidum was the most important of eight FCs identified using sparse classification algorithms, which may be related to clinically important dysfunctions of subjects with StD such as anhedonia and hyposensitivity to rewards. Investigation of whether modulation of the identified FCs can be an effective treatment for StD may be an important future research topic.

## Methods

### Participants

Participants were enrolled from 18 to 19-year-old first-year students attending Hiroshima University, Japan. Beck's Depression Inventory-II (BDI-II)^49^ was administered to the participants to score depressive severity. A BDI-II score of 13 is a suggested cutoff point to detect depression in college students^50,51^; therefore, in the present study, StD was defined as a score of ≥ 13 points on the BDI-II. Participants that met the following criteria after completing the Japanese version of the Composite International Diagnostic Interview^52^ were excluded from this study: major depressive episode during the past year, a lifetime history of bipolar disorder, taking psychopharmacological or psychological treatment within the past year, possibility of acute suicide attempts, difficulty in understanding the purpose of the study, or difficulty in completing the self-report scales due to a serious mental condition or severe physical disease. In this way, a total of 91 subjects were enlisted in the rs-fMRI experiments, including 30 StD subjects with BDI-II scores from 13 to 27 (19 men and 11 women) and 61 age-, sex-, and education-matched healthy controls (HCs) with BDI-II scores < 8 (32 men and 29 women). Demographic data for the participants are presented in Table 1. This study was approved by the ethics committee of Hiroshima University, and all participants provided their written informed consent after the study was completely described to each participant. In addition, we confirm that all procedures were carried out in accordance with relevant guidelines and regulations.

### BDI-II

The BDI-II is a widely-used instrument for measuring depression and depressive symptoms and consists of 21 self-report items that are rated on a 4-point scale ranging from 0 to 3. The Japanese version of the BDI-II has shown good validity and reliability^48^. Additionally, anhedonia was assessed by the BDI-II anhedonic subscore: item #4—loss of pleasure, item #12—loss of interest, item # 15—loss of energy, and item #21—loss of sex drive^53–55^.

### Environmental Reward Observation Scale (EROS)

The EROS^56^ is used to assess environmental reward and response-contingent positive reinforcement. The original version of this assessment is a 10-item scale measuring agreement (1: strongly disagree to 4: strongly agree). The Japanese version of the EROS has shown good validity and reliability^57^. Additionally, this scale shows a negative correlation with depressive symptoms, especially anhedonia symptoms^57^.

### fMRI data acquisition

In a darkened scanning room, the participants were required to maintain their gaze on a fixation point in the center of a monitor screen, not to think of anything specific, and to stay awake. Details of the imaging protocols for fMRI data acquisition and the procedure in each dataset are shown in Supplementary Table S1.

### fMRI data preprocessing

T1-weighted structural images and resting-state functional images were preprocessed with SPM8 (Wellcome Trust Centre for Neuroimaging, University College London, UK) running on MATLAB R2019a (MathWorks, Inc., Natick, MA, USA). The functional images were corrected for slice-timing and subsequently realigned to the mean image. Next, using the associated parameters obtained through the segmentation of the T1-weighted structural images coregistered to the mean functional image, the fMRI data were normalized and resampled in 2 × 2 × 2 mm^3^ voxels. All functional images were then smoothed using an isotropic 6-mm full-width half-maximum Gaussian kernel. Finally, a scrubbing procedure^58^ was used to compensate further for motion, removing any volume (i.e., functional images) with excessive movement (frame displacement > 0.5 mm), based on the relative changes from frame to frame in the fMRI time-series (Supplementary Table S2 for a summary of head motion).

### Interregional correlations

For each participant, pairwise interregional FCs were evaluated between 137 regions of interest, which were defined anatomically using the BrainVISA Sulci Atlas^59,60^ covering the entire cerebral cortex and subcortical regions. Then, the time course of fMRI data in each region was extracted. In this study, the cerebellum was not incorporated in the construction of the classifier because it was truncated in many participants' images. After applying a band-pass filter (transmission range, 0.008–0.1 Hz), the temporal fluctuations of the white matter, cerebrospinal fluid, and entire brain as well as six head motion parameters were linearly regressed out. Then, for each participant, we calculated pairwise Pearson’s correlations among 137 regions of interest in order to generate a matrix of 9316 FCs, which were normalized using Z-score transformation. The scrubbing procedure was employed to remove any frames exhibiting abrupt head motions in the filtered time course.

### Classification algorithm for FC selection

An StD classifier with the identified primary FCs was developed through the combined use of two machine learning algorithms. The procedure for selecting characteristic FCs, training a classifier prediction model, and evaluating its generalizability was performed as a sequential process of 5 × 5 nested feature-selection and leave-one-out cross validation (see the schematic flowchart in Supplementary Fig. S2). As the first algorithm, in order to reduce the number of features to remove the effects of nuisance variables (NVs) including sex and age that may result in catastrophic overfitting, L1-SCCA^23^ was used. L1-SCCA retained FCs that have an association with a canonical variable related only to the Diagnosis label and not to NVs (details are provided in the Supplementary Information of our previous study^25^). As the second algorithm, in order to identify a small number of features with a high contribution to the classifier, SLR^24^ was used. SLR is capable of training a logistic regression model, while objectively deleting FCs that are useless for the purpose of identifying StD. In SLR, the final number of features according to the principle of automatic relevance determination^61^ is decided automatically without manually adjusting the hyper-parameters. On this process approach, SLR classifier was trained by using all-but-one subjects in each leave-one-out (LOO) cross-validation (CV). A sequential process of nested-feature selection and LOOCV is applied to prevent information leakage and over-optimistic results^62^. Nested cross validation and LOOCV were combined in our machine learning algorithm. In order to have more than 20 subjects per fold, we used 5-folds CV for StD classifier development. Classification accuracy was evaluated using the output of the logistic regression classifier generated at the end of the LOOCV procedure. In conclusion, through the combined use of two machine learning algorithms, prior to training SLR, it is essential to reduce the input dimension to a certain extent and remove the effects of NVs that may result in catastrophic overfitting. Therefore, before LOOCV, nested feature-selection was carried out using L1-SCCA. The robustness and stability of the identified FCs across the LOOCV procedure were confirmed by computing their cumulative absolute weights: ${\mathcalligra{c}}^{\kappa } = \sum \nolimits_{\mathfrak{i}=1}^{\mathrm{N}}\left|{\mathcal{W}}_{\mathfrak{i}}^{\kappa }\right|$, where *N* is the number of LOOCV folds (i.e., the number of subjects) and ${\mathcal{W}}_{\mathfrak{i}}^{\kappa }$ is the weight associated with the $\kappa$-th FC during the $\mathfrak{i}$-th LOOCV fold. A greater magnitude of ${\mathcalligra{c}}^{\kappa }$ indicates a more significant contribution by the $\kappa$-th FC to the classification into StD and HC throughout the LOOCV procedure. More details of this algorithm were provided in a study of autism spectrum disorder^22^. The original code is also accessible (please contact the server administrator of the ATR Brain Information Communication Research Laboratory: asd-classifier@atr.jp).

### Generalization to an independent dataset

Participants for an independent validation dataset were recruited from first-year students attending Hiroshima University in a different year, who were diagnosed using the BDI-II and Mini-International Neuropsychiatric Interview^63,64^. These participants met the same inclusion and exclusion criteria as the training dataset (StD subjects: *n* = 16; HCs: *n* = 27, Table 1). Details of fMRI data acquisition are shown in Supplementary Table S1. The MRI data were preprocessed in the same way as for the training dataset, and then the interregional FCs for each subject were calculated. Written informed consent was obtained from all participants before participating in the study. The ethics committee of Hiroshima University approved this study.

### Correlations between the identified FCs and clinical measures

The association between the identified FCs in the StD classifier and clinical assessments relative to anhedonia and reward responsiveness (BDI-II anhedonic subscore and EROS, respectively) was tested. For correlation analyses, Pearson's and Spearman's rank correlation coefficients were calculated when normality was not rejected and was rejected, respectively, using SPSS Statistics 25 software (SPSS, Inc., Chicago, IL, USA). Bonferroni's correction was used to adjust for multiple comparisons, and the significance level was set as *p* < 0.05/8 (= 0.006).

## Supplementary Information

Supplementary Information.

## Data Availability

The datasets generated during and/or analyzed during the current study are available from the corresponding author on reasonable request.
