# Supplementary material for: Resting-state functional connectivity disruption between the left and right pallidum as a biomarker for subthreshold depression
Source: Sci Rep. 2023 Apr 18;13:6349. doi: 10.1038/s41598-023-33077-3 (PMC10113366; doi:10.1038/s41598-023-33077-3)
Supplement: Supplementary file 1 — Supplementary Information. [file 41598_2023_33077_MOESM1_ESM.pdf]

Supplementary material for

Title: Resting-state functional connectivity disruption between the left and right pallidum as a biomarker for subthreshold depression

Yosuke Sato, Go Okada, Satoshi Yokoyama, Naho Ichikawa, Masahiro Takamura, Yuki Mitsuyama, Ayaka Shimizu, Eri Itai, Hotaka Shinzato, Mitsuo Kawato, Noriaki Yahata, Yasumasa Okamoto

Contents

Supplementary Result

Supplementary Figure S1

Supplementary Figure S2

Supplementary Table S1

Supplementary Table S2

Supplementary Table S3

Supplementary Result

We analyzed previous data (92 patients with MDD vs. 92 HCs from Ichikawa et al., 2020) focusing on the FC between bilateral pallidum. As a result, there was a trend toward decreased FC between bilateral pallidum in MDD compared to HCs, which was not significant ($p = 0.067$).

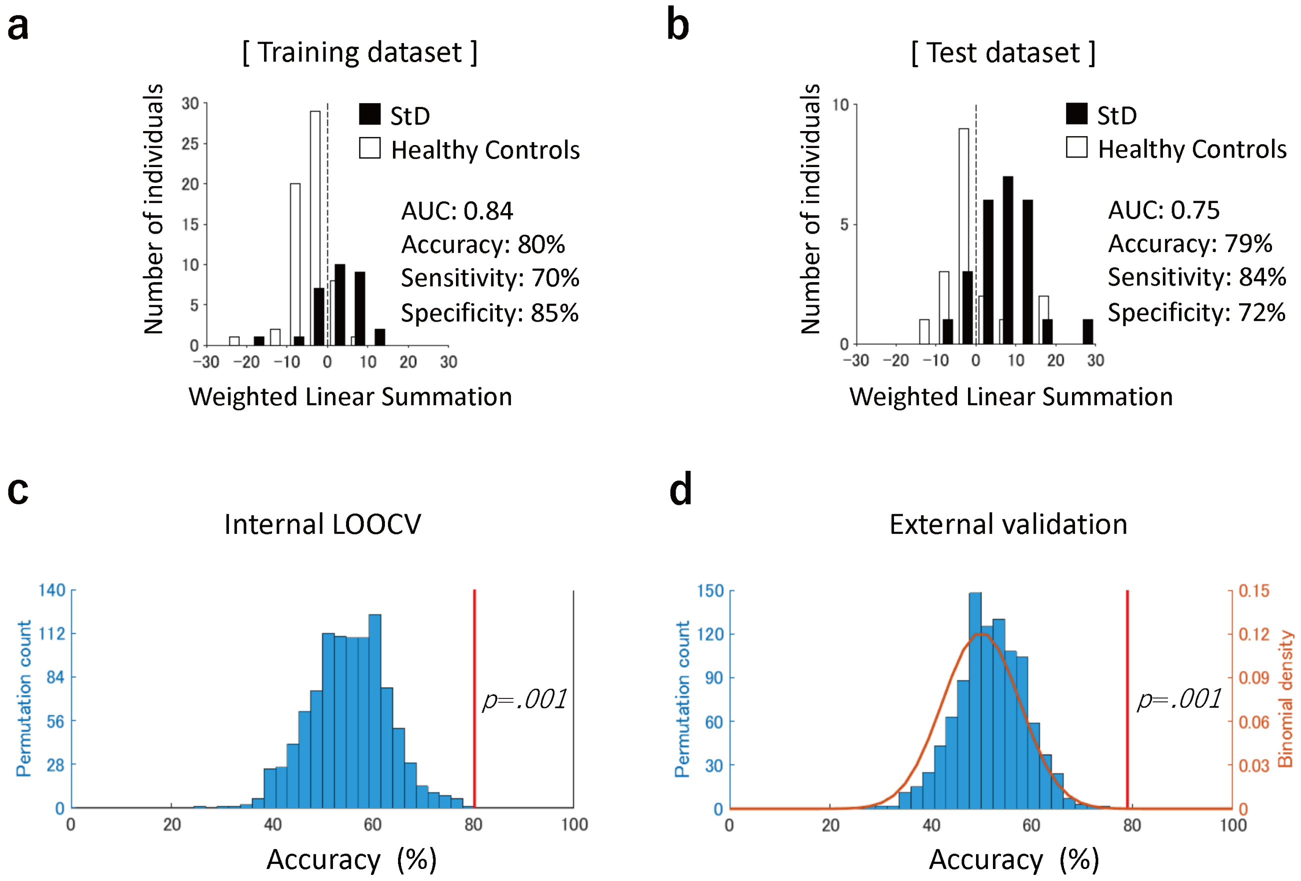

Supplementary Figure S1. Accuracy of the subthreshold depression (StD) classifier.

Distribution of the weighted linear summations of functional connections based on the classification of StD and healthy controls for (a) the training dataset (StD: $n = 30$; healthy controls: $n = 61$) and (b) the test dataset from an independent cohort (StD: $n = 16$; healthy controls: $n = 27$). (c) Histogram of the permutation test (1,000 repetitions) for leave-one-out cross validation (LOOCV) for the training dataset. (d) Histogram of the permutation test (1,000 repetitions) for accuracy in the independent test dataset. The binomial distribution is presented as an orange curve in (d). The accuracy of the StD classifier trained and tested without permutations is presented as vertical red lines in (c–d). LOOCV of the training dataset and independent test dataset was significant at $p = 0.001$, as shown by the results of the permutation test.

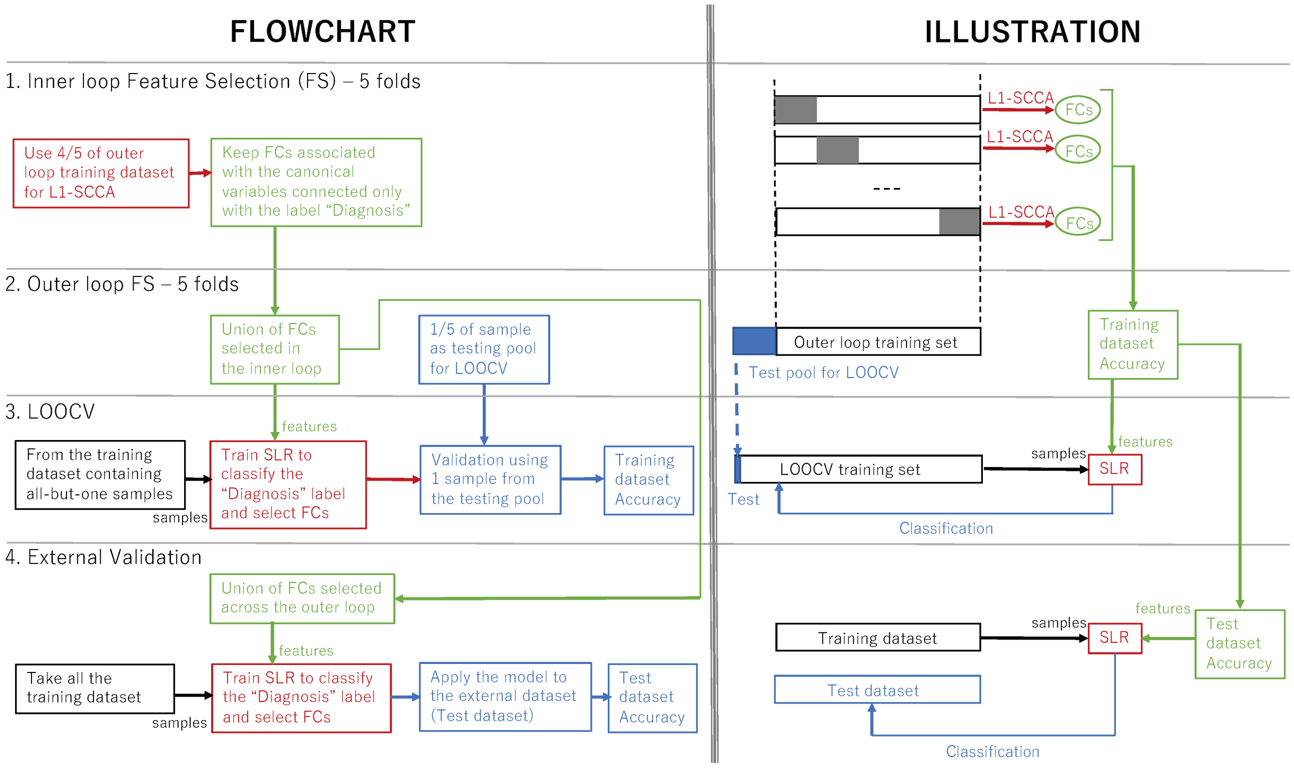

Supplementary Figure S2. Schematic flowchart of the procedure for selecting FCs as StD biomarkers and assessing their predictive power (adapted from Yahata et al., Nat Commun 2016).

The left and right panels represent, respectively, the flowchart and illustration of the procedure. Black, blue, red, and green colors are conceptually associated with training, testing, methods, and features, respectively. (1) In each iteration of the inner loop feature selection (FS), 4/5 of the outer loop training set are used to train L1-SCCA with different hyper-parameters. Functional connectivity features (FCs) that are associated with the canonical variables connected only with the label "Diagnosis" are retained. (2) In the outer loop FS, 1/5 of the samples are retained as testing pool for leave-one-out cross-validation (LOOCV), and the union of the FCs selected throughout the inner loop is derived. (3) One sample is taken from the testing pool of the outer loop, and used as test set of LOOCV. The remaining samples are used to train SLR on the union of the FCs retained during the inner loop. This procedure is repeated for every sample in the testing pool of the outer loop. In this way, the test set of

LOOCV is always independent from the dataset used to select features. (4) The union of the FCs selected across the outer loop is used to train the final SLR on the training dataset, and validated using an external test dataset). In conclusion, nested feature selection is used to remove nuisance FCs, LOOCV is used to quantify generalizability on the training dataset, and the external validation is used to quantify generalizability on the independent dataset (adapted form Yahata et al., Nat Commun 2016).

Supplementary Table S1. Scanner information and resting-state functional magnetic imaging protocols.

Parameter	Training dataset	Test dataset
MRI scanner	Siemens Verio	Siemens Skyra-fit
Participants (StD/healthy controls)	30/61	16/27
Magnetic field (T)	3	3
Field of view (mm)	212	206
Matrix	64 × 64	86 × 86
Number of slices	40	60
Number of volumes	244	375
In-plane resolution (mm)	3.3 × 3.3	2.4 × 2.4
Slice thickness (mm)	3.2	2.4
Slice gap (mm)	0.8	0
TR (ms)	2,500	800
TE (ms)	30	34.4
Total scan time (mm:ss)	10:10	5:08
Flip angle (degrees)	80	52
Slice acquisition order	Ascending	Ascending (interleaved)
Instructions to participants and other imaging conditions	Please relax. Do not think of anything in particular, do not sleep, but keep looking at the crosshair mark. The lights in the scanning room were dimmed.	Same as for the Training dataset

MRI, magnetic resonance imaging; StD, subthreshold depression.

Supplementary Table S2. Head motion of subjects with subthreshold depression (StD) and healthy controls in the training and test datasets.

		Training dataset			Test dataset		
		Healthy controls	StD	*p*	Healthy controls	StD	*p*
Translation	x	0.017 ± 0.010	0.023 ± 0.018	0.054	0.007 ± 0.003	0.007 ± 0.005	0.910
(mm)	y	0.046 ± 0.015	0.038 ± 0.015	0.023	0.039 ± 0.013	0.042 ± 0.016	0.413
	z	0.062 ± 0.034	0.064 ± 0.050	0.798	0.038 ± 0.015	0.051 ± 0.030	0.065
Rotation	x	0.036 ± 0.014	0.038 ± 0.025	0.565	0.025 ± 0.007	0.027 ± 0.008	0.594
(mm)	y	0.012 ± 0.007	0.015 ± 0.014	0.165	0.008 ± 0.003	0.008 ± 0.002	0.797
	z	0.011 ± 0.006	0.014 ± 0.011	0.076	0.006 ± 0.002	0.006 ± 0.003	0.976

Note: head radius was assumed to be 50 mm.

Supplementary Table S3. In the sum of training and test dataset, relationship between each functional correlation value and clinical score.

Identified FCs	BDI anhedonic subscore		EROS	
	r	*p*	*r*	*p*
FC#1	−0.334	0.000*	0.306	0.000*
FC#2	−0.135	0.121	0.136	0.116
FC#3	0.210	0.015	−0. 033	0.706
FC#4	−0.126	0.148	0.063	0.469
FC#5	−0.136	0.118	0.134	0.124
FC#6	−0.033	0.700	0.256	0.003*
FC#7	0.159	0.066	−0.111	0.201
FC#8	−0.094	0.280	0.148	0.087

Note: Clinical scores associated with anhedonia (BDI anhedonic subscore and EROS). *Bonferroni-adjusted significance level of $p < 0.05 / 8$ (=0.006). BDI, Beck's Depression Inventory; EROS, Environmental Reward Observation Scale; FC, functional connection.
